# Di-*n*-butyl­bis(*N*-*n*-butyl-*N*-ethyl­dithio­carbamato-κ*S*)tin(IV)

**DOI:** 10.1107/S1600536809014883

**Published:** 2009-04-30

**Authors:** Ibrahim Baba, Nur Nadia Dzulkefli, Seik Weng Ng

**Affiliations:** a School of Chemical Sciences and Food Technology, Universiti Kebangsaan Malaysia, 43600 Bangi, Selangor Darul Ehsan, Malaysia; b Department of Chemistry, University of Malaya, 50603 Kuala Lumpur, Malaysia

## Abstract

The Sn atom in the title compound, [Sn(C_4_H_9_)_2_(C_7_H_14_NS_2_)_2_], exists in a tetra­hedral C_2_S_2_Sn coordination geometry. The geometry is distorted towards skew-trapezoidal-bipyramidal owing to the proximity of the double-bond S atoms [Sn—S = 2.521 (2) and Sn⋯S = 2.933 (2) Å]. The Sn atom lies on a special position of *mm*2 site symmetry and the tin-bound *n*-butyl chain is disordered about a mirror plane. The ethyl and *n*-butyl groups of the dithio­carbamate unit are disordered about another mirror plane.

## Related literature

For other di-*n*-butyl­tin dithio­carbamates, see: Farina *et al.* (2000 ▶); Lokaj *et al.* (1986 ▶); Menezes *et al.* (2005 ▶); Vrábel *et al.* (1992*a* ▶,*b* ▶); Vrábel & Kellö (1993 ▶); Zia-ur-Rehman *et al.* (2006 ▶). For a review of the applications and structures of tin dithio­carbamates, see: Tiekink (2008 ▶).
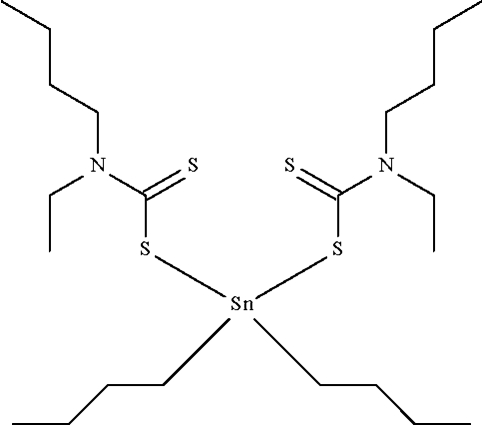


## Experimental

### 

#### Crystal data

[Sn(C_4_H_9_)_2_(C_7_H_14_NS_2_)_2_]
*M* *_r_* = 585.54
Orthorhombic, 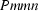

*a* = 11.1317 (2) Å
*b* = 19.4349 (3) Å
*c* = 7.7262 (1) Å
*V* = 1671.51 (5) Å^3^
*Z* = 2
Mo *K*α radiation
μ = 1.02 mm^−1^
*T* = 123 K
0.30 × 0.25 × 0.20 mm

#### Data collection

Bruker SMART APEX diffractometer
Absorption correction: multi-scan (*SADABS*; Sheldrick, 1996 ▶) *T*_min_ = 0.749, *T*_max_ = 0.821
11224 measured reflections
2072 independent reflections
1667 reflections with *I* > 2σ(*I*)
*R*_int_ = 0.024

#### Refinement

*R*[*F* ^2^ > 2σ(*F* ^2^)] = 0.066
*wR*(*F* ^2^) = 0.239
*S* = 1.11
2072 reflections
131 parameters
55 restraints
H-atom parameters constrained
Δρ_max_ = 1.03 e Å^−3^
Δρ_min_ = −0.66 e Å^−3^

### 

Data collection: *APEX2* (Bruker, 2008 ▶); cell refinement: *SAINT* (Bruker, 2008 ▶); data reduction: *SAINT*; program(s) used to solve structure: *SHELXS97* (Sheldrick, 2008 ▶); program(s) used to refine structure: *SHELXL97* (Sheldrick, 2008 ▶); molecular graphics: *X-SEED* (Barbour, 2001 ▶); software used to prepare material for publication: *publCIF* (Westrip, 2009 ▶).

## Supplementary Material

Crystal structure: contains datablocks global, I. DOI: 10.1107/S1600536809014883/tk2428sup1.cif

Structure factors: contains datablocks I. DOI: 10.1107/S1600536809014883/tk2428Isup2.hkl

Additional supplementary materials:  crystallographic information; 3D view; checkCIF report

## Figures and Tables

**Fig. 1.**
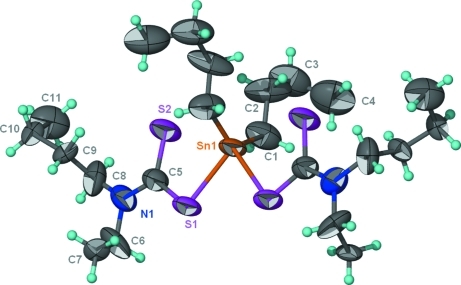
Thermal ellipsoid plot (Barbour, 2001) of dibutyldi(N-butyl-N-ethylbutyldithiocarbamato)tin at the 70% probability level; the disorder is not shown. Unlabelled atoms are related by a 2-fold axis. Hydrogen atoms are drawn as spheres of arbitrary radius.

## supplementary crystallographic information

### Experimental

Carbon disulfide (4 ml, 0.06 mol) was added to *n*-butylisopropylamine (8 ml, 0.06 mol) in ethanol (50 ml) at 277 K. Dibutyltin dichloride (9.1 g, 0.03 mol) dissolved in ethanol (50 ml) was added. The white solid that precipitated was collected and recrystallized from ethanol.

### Refinement

The tin-bound butyl chain was allowed to refined off the mirror plane, as were the ethyl and butyl groups of the dithiocarbamate anion. 1,2-Related carbon-carbon distances were restrained to 1.54±0.01 Å and the 1,3-related ones to 2.51±0.02 Å. The N1–C6 and N1–C6' pair of distances were restrained to 0.01 Å as were the N1–C8 and N1–C8' pair. The temperature factors of the primed atoms were restrained to those of the unprimed ones; the anisotropic displacement parameters of the primed atoms were restrained to be nearly isotropic.

Carbon-bound H-atoms were placed in calculated positions (C—H 0.95 to 0.99 Å) and were included in the refinement in the riding model approximation, with *U*(H) set to 1.2 to 1.5*U*(C). The final difference Fourier map had a large peak in the vicinity of the C9' atom.

### Crystal data

**Table:**

[Sn(C_4_H_9_)_2_(C_7_H_14_NS_2_)_2_]	*F*(000) = 612
*M**_r_* = 585.54	*D*_x_ = 1.163 Mg m^−^^3^
Orthorhombic, *P**m**m**n*	Mo *K*α radiation, λ = 0.71073 Å
Hall symbol: -P 2ab 2a	Cell parameters from 5407 reflections
*a* = 11.1317 (2) Å	θ = 2.8–28.2°
*b* = 19.4349 (3) Å	µ = 1.02 mm^−^^1^
*c* = 7.7262 (1) Å	*T* = 123 K
*V* = 1671.51 (5) Å^3^	Block, colorless
*Z* = 2	0.30 × 0.25 × 0.20 mm

### Data collection

**Table:**

Bruker SMART APEX diffractometer	2072 independent reflections
Radiation source: fine-focus sealed tube	1667 reflections with *I* > 2σ(*I*)
graphite	*R*_int_ = 0.024
ω scans	θ_max_ = 27.5°, θ_min_ = 2.1°
Absorption correction: multi-scan (*SADABS*; Sheldrick, 1996)	*h* = −14→14
*T*_min_ = 0.749, *T*_max_ = 0.821	*k* = −24→25
11224 measured reflections	*l* = −10→9

### Refinement

**Table:**

Refinement on *F*^2^	Primary atom site location: structure-invariant direct methods
Least-squares matrix: full	Secondary atom site location: difference Fourier map
*R*[*F*^2^ > 2σ(*F*^2^)] = 0.066	Hydrogen site location: inferred from neighbouring sites
*wR*(*F*^2^) = 0.239	H-atom parameters constrained
*S* = 1.11	*w* = 1/[σ^2^(*F*_o_^2^) + (0.1484*P*)^2^ + 2.8257*P*] where *P* = (*F*_o_^2^ + 2*F*_c_^2^)/3
2072 reflections	(Δ/σ)_max_ = 0.001
131 parameters	Δρ_max_ = 1.03 e Å^−^^3^
55 restraints	Δρ_min_ = −0.66 e Å^−^^3^

### Fractional atomic coordinates and isotropic or equivalent isotropic displacement parameters (Å^2^)

**Table:**

	*x*	*y*	*z*	*U*_iso_*/*U*_eq_	Occ. (<1)
Sn1	0.7500	0.2500	0.43290 (7)	0.0602 (4)	
S1	0.7500	0.33480 (11)	0.6798 (2)	0.0659 (6)	
S2	0.7500	0.39534 (11)	0.3309 (3)	0.0852 (9)	
N1	0.7500	0.4700 (4)	0.6210 (11)	0.0661 (18)	
C1	0.5831 (12)	0.257 (4)	0.3153 (12)	0.085 (10)	0.50
H1A	0.5315	0.2190	0.3580	0.102*	0.50
H1B	0.5448	0.3008	0.3488	0.102*	0.50
C2	0.5905 (11)	0.2529 (13)	0.1171 (11)	0.100 (5)	0.50
H2A	0.6437	0.2901	0.0752	0.120*	0.50
H2B	0.6277	0.2085	0.0843	0.120*	0.50
C3	0.4697 (14)	0.2591 (17)	0.0264 (19)	0.107 (9)	0.50
H3A	0.4814	0.2581	−0.1006	0.129*	0.50
H3B	0.4314	0.3034	0.0573	0.129*	0.50
C4	0.3882 (18)	0.1990 (16)	0.082 (3)	0.130 (9)	0.50
H4A	0.3075	0.2058	0.0345	0.195*	0.50
H4B	0.3839	0.1973	0.2087	0.195*	0.50
H4C	0.4215	0.1557	0.0384	0.195*	0.50
C5	0.7500	0.4065 (5)	0.5500 (10)	0.0576 (18)	
C6	0.744 (3)	0.4806 (13)	0.8169 (16)	0.085 (4)	0.25
H6A	0.7181	0.5282	0.8426	0.102*	0.25
H6B	0.6849	0.4485	0.8678	0.102*	0.25
C7	0.865 (3)	0.4679 (13)	0.8947 (16)	0.049 (4)	0.25
H7A	0.8610	0.4755	1.0200	0.074*	0.25
H7B	0.9235	0.4996	0.8437	0.074*	0.25
H7C	0.8896	0.4204	0.8716	0.074*	0.25
C6'	0.732 (3)	0.4781 (19)	0.8168 (18)	0.085 (4)	0.25
H6'1	0.6980	0.4354	0.8668	0.102*	0.25
H6'2	0.6764	0.5166	0.8415	0.102*	0.25
C7'	0.857 (3)	0.4929 (13)	0.895 (3)	0.049 (4)	0.25
H7'1	0.8499	0.4994	1.0199	0.074*	0.25
H7'2	0.8904	0.5346	0.8419	0.074*	0.25
H7'3	0.9108	0.4539	0.8711	0.074*	0.25
C8	0.7479 (19)	0.5360 (8)	0.521 (3)	0.083 (4)	0.50
H8A	0.7211	0.5739	0.5971	0.100*	0.25
H8B	0.6901	0.5321	0.4240	0.100*	0.25
C9	0.8705 (19)	0.5520 (8)	0.451 (3)	0.047 (4)	0.25
H9A	0.8942	0.5147	0.3706	0.056*	0.25
H9B	0.9283	0.5519	0.5483	0.056*	0.25
C10	0.8813 (14)	0.6206 (8)	0.356 (3)	0.042 (3)	0.25
H10A	0.9489	0.6186	0.2727	0.050*	0.25
H10B	0.8979	0.6579	0.4398	0.050*	0.25
C11	0.764 (3)	0.6359 (14)	0.259 (4)	0.102 (6)	0.25
H11A	0.7647	0.6837	0.2178	0.153*	0.25
H11B	0.6955	0.6291	0.3365	0.153*	0.25
H11C	0.7567	0.6048	0.1592	0.153*	0.25
C8'	0.734 (3)	0.5340 (12)	0.513 (5)	0.083 (4)	0.25
H8C	0.6770	0.5655	0.5725	0.100*	0.25
H8D	0.6985	0.5215	0.3999	0.100*	0.25
C9'	0.8539 (19)	0.5710 (11)	0.485 (3)	0.047 (4)	0.25
H9C	0.8873	0.5851	0.5982	0.056*	0.25
H9D	0.9114	0.5388	0.4302	0.056*	0.25
C10'	0.8399 (14)	0.6346 (8)	0.370 (3)	0.042 (3)	0.25
H10C	0.8562	0.6218	0.2482	0.050*	0.25
H10D	0.8999	0.6696	0.4046	0.050*	0.25
C11'	0.7130 (18)	0.6661 (16)	0.383 (4)	0.102 (6)	0.25
H11D	0.7018	0.6997	0.2898	0.153*	0.25
H11E	0.7037	0.6889	0.4951	0.153*	0.25
H11F	0.6528	0.6295	0.3717	0.153*	0.25

### Atomic displacement parameters (Å^2^)

**Table:**

	*U*^11^	*U*^22^	*U*^33^	*U*^12^	*U*^13^	*U*^23^
Sn1	0.1059 (7)	0.0502 (5)	0.0245 (4)	0.000	0.000	0.000
S1	0.1097 (17)	0.0588 (11)	0.0292 (8)	0.000	0.000	−0.0063 (7)
S2	0.172 (3)	0.0471 (11)	0.0367 (9)	0.000	0.000	−0.0001 (8)
N1	0.080 (4)	0.055 (4)	0.064 (4)	0.000	0.000	−0.018 (3)
C1	0.112 (9)	0.08 (3)	0.062 (6)	0.03 (2)	−0.015 (6)	−0.007 (11)
C2	0.135 (11)	0.112 (11)	0.053 (5)	0.075 (14)	0.011 (7)	0.029 (11)
C3	0.145 (13)	0.11 (2)	0.070 (7)	0.041 (19)	−0.030 (9)	0.006 (12)
C4	0.137 (18)	0.16 (2)	0.089 (13)	0.024 (18)	−0.032 (12)	0.002 (13)
C5	0.073 (5)	0.057 (4)	0.043 (4)	0.000	0.000	−0.006 (3)
C6	0.086 (8)	0.091 (6)	0.077 (6)	0.000 (8)	−0.029 (8)	−0.042 (5)
C7	0.048 (5)	0.066 (11)	0.034 (4)	0.002 (9)	−0.017 (4)	0.016 (6)
C6'	0.086 (8)	0.091 (6)	0.077 (6)	0.000 (8)	−0.029 (8)	−0.042 (5)
C7'	0.048 (5)	0.066 (11)	0.034 (4)	0.002 (9)	−0.017 (4)	0.016 (6)
C8	0.068 (7)	0.063 (5)	0.118 (7)	−0.021 (8)	0.010 (9)	0.001 (5)
C9	0.040 (6)	0.057 (8)	0.044 (7)	0.002 (6)	0.011 (5)	0.013 (7)
C10	0.031 (8)	0.035 (6)	0.058 (6)	0.010 (5)	0.014 (6)	0.005 (5)
C11	0.119 (11)	0.092 (9)	0.095 (9)	0.039 (9)	−0.007 (8)	0.002 (7)
C8'	0.068 (7)	0.063 (5)	0.118 (7)	−0.021 (8)	0.010 (9)	0.001 (5)
C9'	0.040 (6)	0.057 (8)	0.044 (7)	0.002 (6)	0.011 (5)	0.013 (7)
C10'	0.031 (8)	0.035 (6)	0.058 (6)	0.010 (5)	0.014 (6)	0.005 (5)
C11'	0.119 (11)	0.092 (9)	0.095 (9)	0.039 (9)	−0.007 (8)	0.002 (7)

### Geometric parameters (Å, °)

**Table:**

Sn1—C1	2.073 (13)	C7—H7B	0.9800
Sn1—C1^i^	2.073 (13)	C7—H7C	0.9800
Sn1—C1^ii^	2.073 (13)	C6'—C7'	1.545 (10)
Sn1—C1^iii^	2.073 (13)	C6'—H6'1	0.9900
Sn1—S1	2.5211 (19)	C6'—H6'2	0.9900
Sn1—S1^iii^	2.5211 (19)	C7'—H7'1	0.9800
S1—C5	1.718 (9)	C7'—H7'2	0.9800
S2—C5	1.707 (8)	C7'—H7'3	0.9800
N1—C5	1.350 (11)	C8—C9	1.4998
N1—C8	1.499 (14)	C8—H8A	0.9900
N1—C8'	1.508 (15)	C8—H8B	0.9900
N1—C8'^ii^	1.508 (15)	C9—C10	1.527 (9)
N1—C6	1.528 (14)	C9—H9A	0.9900
N1—C6^ii^	1.528 (14)	C9—H9B	0.9900
N1—C6'^ii^	1.534 (15)	C10—C11	1.536 (10)
N1—C6'	1.534 (15)	C10—H10A	0.9900
C1—C2	1.536 (9)	C10—H10B	0.9900
C1—H1A	0.9900	C11—H11A	0.9800
C1—H1B	0.9900	C11—H11B	0.9800
C2—C3	1.520 (9)	C11—H11C	0.9800
C2—H2A	0.9900	C8'—C9'	1.536 (10)
C2—H2B	0.9900	C8'—H8C	0.9900
C3—C4	1.540 (10)	C8'—H8D	0.9900
C3—H3A	0.9900	C9'—C10'	1.529 (10)
C3—H3B	0.9900	C9'—H9C	0.9900
C4—H4A	0.9800	C9'—H9D	0.9900
C4—H4B	0.9800	C10'—C11'	1.543 (10)
C4—H4C	0.9800	C10'—H10C	0.9900
C6—C7	1.4925	C10'—H10D	0.9900
C6—H6A	0.9900	C11'—H11D	0.9800
C6—H6B	0.9900	C11'—H11E	0.9800
C7—H7A	0.9800	C11'—H11F	0.9800
			
C1—Sn1—C1^ii^	127.4 (9)	C6—C7—H7A	109.5
C1^i^—Sn1—C1^ii^	128.0 (6)	C6—C7—H7B	109.5
C1—Sn1—C1^iii^	128.0 (6)	H7A—C7—H7B	109.5
C1^i^—Sn1—C1^iii^	127.4 (9)	C6—C7—H7C	109.5
C1—Sn1—S1	106.9 (14)	H7A—C7—H7C	109.5
C1^i^—Sn1—S1	111.9 (15)	H7B—C7—H7C	109.5
C1^ii^—Sn1—S1	106.9 (14)	N1—C6'—C7'	106.5 (19)
C1^iii^—Sn1—S1	111.9 (15)	N1—C6'—H6'1	110.4
C1—Sn1—S1^iii^	111.9 (15)	C7'—C6'—H6'1	110.4
C1^i^—Sn1—S1^iii^	106.9 (14)	N1—C6'—H6'2	110.4
C1^ii^—Sn1—S1^iii^	111.9 (15)	C7'—C6'—H6'2	110.4
C1^iii^—Sn1—S1^iii^	106.9 (14)	H6'1—C6'—H6'2	108.6
S1—Sn1—S1^iii^	81.64 (10)	C6'—C7'—H7'1	109.5
C5—S1—Sn1	95.1 (3)	C6'—C7'—H7'2	109.5
C5—N1—C8	124.9 (12)	H7'1—C7'—H7'2	109.5
C5—N1—C8'	122.0 (17)	C6'—C7'—H7'3	109.5
C5—N1—C8'^ii^	122.0 (17)	H7'1—C7'—H7'3	109.5
C5—N1—C6	121.7 (12)	H7'2—C7'—H7'3	109.5
C8—N1—C6	113.3 (14)	N1—C8—C9	110.5 (8)
C8'—N1—C6	115.5 (18)	N1—C8—H8A	109.6
C8'^ii^—N1—C6	116.1 (19)	C9—C8—H8A	109.6
C5—N1—C6^ii^	121.7 (12)	N1—C8—H8B	109.6
C8—N1—C6^ii^	113.4 (14)	C9—C8—H8B	109.6
C8'—N1—C6^ii^	116.1 (19)	H8A—C8—H8B	108.1
C8'^ii^—N1—C6^ii^	115.5 (18)	C8—C9—C10	115.2 (9)
C5—N1—C6'^ii^	119.7 (16)	C8—C9—H9A	108.5
C8—N1—C6'^ii^	115.1 (18)	C10—C9—H9A	108.5
C8'—N1—C6'^ii^	118 (2)	C8—C9—H9B	108.5
C8'^ii^—N1—C6'^ii^	116 (2)	C10—C9—H9B	108.5
C5—N1—C6'	119.7 (16)	H9A—C9—H9B	107.5
C8—N1—C6'	114.8 (18)	C9—C10—C11	109.7 (12)
C8'—N1—C6'	116 (2)	C9—C10—H10A	109.7
C8'^ii^—N1—C6'	118 (2)	C11—C10—H10A	109.7
C2—C1—Sn1	112.7 (9)	C9—C10—H10B	109.7
C2—C1—H1A	109.1	C11—C10—H10B	109.7
Sn1—C1—H1A	109.1	H10A—C10—H10B	108.2
C2—C1—H1B	109.1	C10—C11—H11A	109.5
Sn1—C1—H1B	109.1	C10—C11—H11B	109.5
H1A—C1—H1B	107.8	H11A—C11—H11B	109.5
C3—C2—C1	114.1 (9)	C10—C11—H11C	109.5
C3—C2—H2A	108.7	H11A—C11—H11C	109.5
C1—C2—H2A	108.7	H11B—C11—H11C	109.5
C3—C2—H2B	108.7	N1—C8'—C9'	111.1 (17)
C1—C2—H2B	108.7	N1—C8'—H8C	109.4
H2A—C2—H2B	107.6	C9'—C8'—H8C	109.4
C2—C3—C4	109.4 (13)	N1—C8'—H8D	109.4
C2—C3—H3A	109.8	C9'—C8'—H8D	109.4
C4—C3—H3A	109.8	H8C—C8'—H8D	108.0
C2—C3—H3B	109.8	C10'—C9'—C8'	111.9 (11)
C4—C3—H3B	109.8	C10'—C9'—H9C	109.2
H3A—C3—H3B	108.2	C8'—C9'—H9C	109.2
C3—C4—H4A	109.5	C10'—C9'—H9D	109.2
C3—C4—H4B	109.5	C8'—C9'—H9D	109.2
H4A—C4—H4B	109.5	H9C—C9'—H9D	107.9
C3—C4—H4C	109.5	C9'—C10'—C11'	112.1 (12)
H4A—C4—H4C	109.5	C9'—C10'—H10C	109.2
H4B—C4—H4C	109.5	C11'—C10'—H10C	109.2
N1—C5—S2	121.3 (7)	C9'—C10'—H10D	109.2
N1—C5—S1	120.3 (6)	C11'—C10'—H10D	109.2
S2—C5—S1	118.4 (5)	H10C—C10'—H10D	107.9
C7—C6—N1	109.8 (11)	C10'—C11'—H11D	109.5
C7—C6—H6A	109.7	C10'—C11'—H11E	109.5
N1—C6—H6A	109.7	H11D—C11'—H11E	109.5
C7—C6—H6B	109.7	C10'—C11'—H11F	109.5
N1—C6—H6B	109.7	H11D—C11'—H11F	109.5
H6A—C6—H6B	108.2	H11E—C11'—H11F	109.5
			
C1—Sn1—S1—C5	−69.5 (16)	C6'^ii^—N1—C5—S1	8.7 (14)
C1^i^—Sn1—S1—C5	−75.1 (16)	C6'—N1—C5—S1	−8.7 (14)
C1^ii^—Sn1—S1—C5	69.5 (16)	Sn1—S1—C5—N1	180.000 (3)
C1^iii^—Sn1—S1—C5	75.1 (16)	Sn1—S1—C5—S2	0.000 (2)
S1^iii^—Sn1—S1—C5	180.000 (2)	C5—N1—C6—C7	78.1 (9)
C8—N1—C5—S2	1.1 (10)	C8—N1—C6—C7	−105.3 (10)
C8'—N1—C5—S2	8.2 (12)	C8'—N1—C6—C7	−112.2 (12)
C8'^ii^—N1—C5—S2	−8.2 (12)	C8'^ii^—N1—C6—C7	−96.8 (13)
C6—N1—C5—S2	177.2 (14)	C6^ii^—N1—C6—C7	−13.4 (4)
C6^ii^—N1—C5—S2	−177.2 (14)	C5—N1—C8—C9	−78.2 (8)
C6'^ii^—N1—C5—S2	−171.3 (14)	C8'—N1—C8—C9	−144 (19)
C6'—N1—C5—S2	171.3 (14)	C8'^ii^—N1—C8—C9	−6(15)
C8—N1—C5—S1	−178.9 (10)	C6—N1—C8—C9	105.4 (14)
C8'—N1—C5—S1	−171.8 (12)	C6^ii^—N1—C8—C9	100.2 (14)
C8'^ii^—N1—C5—S1	171.8 (12)	C6'^ii^—N1—C8—C9	94.5 (15)
C6—N1—C5—S1	−2.8 (14)	C6'—N1—C8—C9	111.1 (14)
C6^ii^—N1—C5—S1	2.8 (14)		

Symmetry codes: (i) *x*, −*y*+1/2, *z*; (ii) −*x*+3/2, *y*, *z*; (iii) −*x*+3/2, −*y*+1/2, *z*.
